# Tool State Recognition Based on POGNN-GRU under Unbalanced Data

**DOI:** 10.3390/s24165433

**Published:** 2024-08-22

**Authors:** Weiming Tong, Jiaqi Shen, Zhongwei Li, Xu Chu, Wenqi Jiang, Liguo Tan

**Affiliations:** 1 Laboratory for Space Environment and Physical Sciences, Harbin Institute of Technology, Harbin 150001, China; tanliguo@hit.edu.cn; 2 School of Electrical Engineering and Automation, Harbin Institute of Technology, Harbin 150001, China; shenjiaqi315@163.com (J.S.); lzw@hit.edu.cn (Z.L.); 20b906062@stu.hit.edu.cn (X.C.); jiangwenqi1997@gmail.com (W.J.)

**Keywords:** state recognition, unbalanced data, graph neural networks, pruned optimized graph

## Abstract

Accurate recognition of tool state is important for maximizing tool life. However, the tool sensor data collected in real-life scenarios has unbalanced characteristics. Additionally, although graph neural networks (GNNs) show excellent performance in feature extraction in the spatial dimension of data, it is difficult to extract features in the temporal dimension efficiently. Therefore, we propose a tool state recognition method based on the Pruned Optimized Graph Neural Network-Gated Recurrent Unit (POGNN-GRU) under unbalanced data. Firstly, design the Improved-Majority Weighted Minority Oversampling Technique (IMWMOTE) by introducing an adaptive noise removal strategy and improving the MWMOTE to alleviate the unbalanced problem of data. Subsequently, propose a POG graph data construction method based on a multi-scale multi-metric basis and a Gaussian kernel weight function to solve the problem of one-sided description of graph data under a single metric basis. Then, construct the POGNN-GRU model to deeply mine the spatial and temporal features of the data to better identify the state of the tool. Finally, validation and ablation experiments on the PHM 2010 and HMoTP datasets show that the proposed method outperforms the other models in terms of identification, and the highest accuracy improves by 1.62% and 1.86% compared with the corresponding optimal baseline model.

## 1. Introduction

In intelligent manufacturing, the condition of the tool has a decisive influence on the quality of product machining. Currently, operators usually rely on their personal experience to decide when to change tools in order to prevent workpiece damage due to excessive tool wear. However, this practice often leads to unnecessary waste of resources, so it is important to monitor the condition of the tool to identify the tool wear status in time and take appropriate measures [1]. Machine vision-based tool condition recognition methods can achieve accurate quantification of tool wear, but their application is usually accompanied by interruptions in the production process, thus negatively affecting productivity. To solve this problem, researchers have proposed deploying sensors near the cutting area and identifying the tool condition through data-driven techniques, thus ensuring the continuity and efficiency of the production process [2,3]. For example, reference [4] combined the tool wear law and its sensor data to construct Siamese Long Short-term Memory Networks (SLSTMs) for tool wear state monitoring. Reference [5] used an improved residual network to extract features from the force and vibration sensor data of the cutting tool, thereby effectively identifying the degree of tool wear. In addition, by analyzing the wear change curve of the tool, the wear state of the tool can be classified into three types: initial wear, normal wear, and severe wear [6]. In the actual production process, the time that the tool is in the normal wear state is much longer than the time that it is in the initial wear state or the severe wear state, resulting in an imbalance in the distribution of sensor data collected in different states. The existing models are mostly designed based on the assumption that data are balanced, and these state monitoring models based on balanced data are prone to producing decision boundaries that are biased towards balanced categories, thus failing to accurately differentiate between state categories in the face of actual unbalanced data and reducing the generalization ability of the model [7]. Therefore, it is of great significance to study the tool state recognition problem under unbalanced data. In recent years, many scholars have conducted extensive research on it and proposed techniques such as variational autoencoder, oversampling, etc., to achieve data volume expansion or data quality improvement, thus effectively alleviating the data imbalance problem [8,9]. Among them, the oversampling techniques represented by SMOTE (Synthetic Minority Over Sampling Technique), BSMOTE (Borderline-SMOTE), Adaptive Synthetic Sampling (ADASYN), and Majority Weighted Minority Oversampling Technique (MWMOTE) can achieve data balance by synthesizing more samples while retaining a large number of samples containing important information. In addition, the above oversampling techniques can effectively alleviate the problems of traditional oversampling techniques due to the generation of overlapping samples or noisy samples, such as unclear decision boundaries or overfitting [10,11,12,13]. For example, Liu et al. [14] adaptively generated many non-repeating samples with the help of ADASYN, which enabled the synthesis of more training data from the minority classes of samples that are more difficult to learn in order to alleviate the data imbalance problem. Wei et al. [15] proposed the Cluster-MWMOTE algorithm to implement the MWMOTE oversampling for the minority class sub-clusters and eliminated the noisy data during data processing, which effectively solved the interclass and intraclass imbalance problem of the bearing data during fault diagnosis. In addition, Zhu et al. [16] improved the distribution of different class samples by synthesizing minority class samples via Borderline SMOTE and focusing on special boundary samples, thus reducing the possibility of overfitting the model and improving the accuracy of fault diagnosis.

On the other hand, most of the existing methods for tool wear state recognition are implemented using artificial intelligence techniques represented by deep learning. For example, Zhou et al. [17] extracted the tool wear characteristics from sensor data with the help of long and short-term memory networks to determine the state in which the tool is in so as to ensure the quality of machined products. Wang et al. [18] improved the accuracy of tool state monitoring by constructing a deep heterogeneous GRU model to achieve the wear characteristics of the tool over a time series. Zhu et al. [19] proposed a CNN-LSTM model to analyze tool images for tool state recognition. Although the above methods can effectively capture the potential features of conventional data, such as time series, they are mostly limited to the features of a single data dimension. In practical scenarios where multiple sensors are usually used to monitor the tool state, ignoring the correlation between the sensor data may make the features extracted from the model one-sided, which may result in the model not being able to reliably identify the real tool state. To further improve the accuracy and reliability of the model, more and more scholars take the interdependence between each sensor data as a reference factor for state recognition and represent it with irregular graphs. In the graph data, edge connectivity relationships and edge weights are often determined by metric such as distance or similarity to reflect the relationships between nodes and their strengths [20,21,22]. However, features such as irregular topology, lack of translation invariance, and complex dependencies between nodes and edges of graph data make them difficult to model in non-Euclidean spaces. In order to solve this problem, researchers have gradually applied graph neural networks (GNNs) to the field of state recognition to achieve the modeling of the interdependence between the data of each sensor and embed it into the extracted features to improve feature extraction and noise immunity [23]. Li et al. [24] established a practical guide for a novel intelligent fault diagnosis and prediction framework based on GNNs, in which three types of graph construction methods are provided and GNNs under seven different graph pooling methods are investigated, and finally these GNNs are benchmarked on a fault diagnosis dataset and a prognostic dataset. Wang et al. [25] proposed a hierarchical Fast Fourier Transform-Long Short-term Memory Networks-Graph Convolutional Networks (FFT-LSTM-GCN) model for fault diagnosis in nuclear power plants. Lin et al. [26] constructed a KGRU hybrid network to analyze spatio-temporal signal variations for fault sensor detection by combining the Multi-Graph Convolutional Network (MGCN) and GRU. Li et al. [27] constructed a distributed graph-attentive bi-directional long and short-term memory model for industrial process global and local fault feature extraction.

By analyzing the above research status, it can be found that in the data processing stage, some of the existing oversampling techniques can effectively alleviate the problem of data imbalance to a certain extent, but they still face problems such as noise sensitivity and easy overfitting. In addition, although some methods can alleviate the above problems, they tend to ignore the local distribution characteristics of the samples and fail to adaptively determine the number of samples to be synthesized for each sample cluster, thus affecting the generalization ability of the model. In the feature extraction stage, graph data construction is crucial for the performance of GNN. Currently, the connection relationship between graph nodes is mostly obtained based on a single index such as distance and probability, such as the literature [23] based on Euclidean distance to construct an undirected weighted *k*-nearest neighbor graph as the input of the Semi-Supervised Graph Convolutional Network (SSGCN). Such methods, although simple and interpretable, may not accurately reflect the real distribution of the data and cannot comprehensively capture the multilevel associations between the data, which leads to problems such as insufficient characterization of feature diversity and a lack of robustness in the constructed graph data. In addition, sensor data are interdependent in both spatial and temporal dimensions, and the lack of spatial and temporal dimensions and their correlation analysis will lead to the tool condition monitoring model not being able to deeply excavate the spatial and temporal characteristics of the tool condition data, thus affecting the ability to influence the model’s accurate judgment and early warning of the tool condition. Therefore, to address the above problems, this paper proposes a state recognition method for tools under unbalanced data. Firstly, an adaptive noise removal strategy is introduced, and the Improved- Majority Weighted Minority Oversampling Technique (IMWMOTE) algorithm is designed based on ADASYN and MWMOTE ideograms to achieve data enhancement for a few classes of samples to alleviate the problem of data imbalance. On this basis, the multi-scale graph is constructed based on different metrics and thresholds, and then pruning optimization and weight calculation of graph node edge relationships are performed based on the structural information of different base graphs combined with the Gaussian kernel weight function, and based on which, different graph data are constructed in the temporal dimension to adequately portray the spatial and temporal correlation of device sensor data. Subsequently, the Pruned Optimized Graph Neural Network-Gated Recurrent Unit (POGNN-GRU) model is constructed to mine the implicit wear state information, so as to realize the intelligent state recognition of the tool. The main contributions of this paper are as follows:

- An IMWMOTE unbalanced fault data processing algorithm is proposed, which can adaptively remove the noise in the minority class fault samples to avoid introducing wrong samples and expanding the inter-class overlap and, at the same time, reasonably estimate the number of samples that need to be synthesized, which can effectively alleviate the unbalance fault data between classes and within classes.
- A graph data construction method based on a multi-scale multi-metric basis and a Gaussian kernel weight function is proposed to obtain more comprehensive POG graph data by optimizing the structural information of the graph data, which improves the representation of topological structure.
- The proposed IMWMOTE and graph data construction methods are used to process the multi-sensor data, on which the POGNN-GRU model is constructed to achieve the collaborative extraction of spatio-temporal features of the data, and the validity and robustness of the method are successfully verified on the PHM 2010 and HMoTP datasets.

The subsequent parts of this paper are as follows: Section 2 describes the problems that may be encountered during the process of tool state recognition and presents the model framework for tool state recognition and the implementation of each part. Section 3 describes the used dataset and experimental results in this study and analyses and evaluates the performance of the tool state recognition model. Finally, the main conclusions obtained from this study are summarized in Section 4.

## 2. Methods

### 2.1. Problem Description and Framework Design

**Figure 1:**
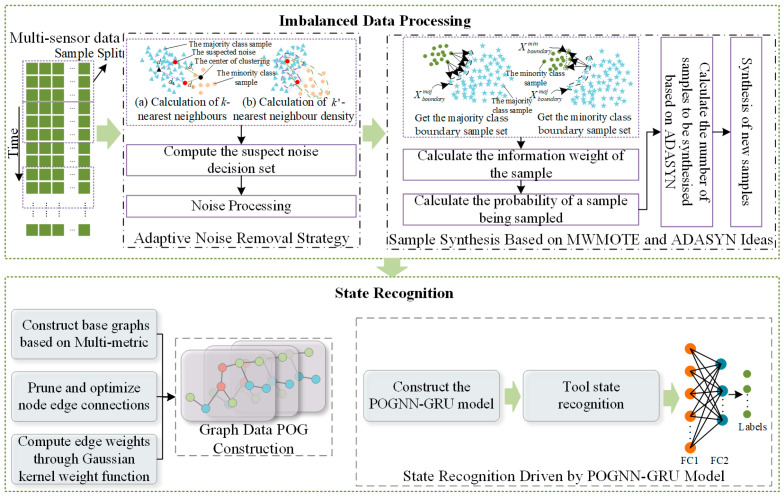
A POGNN-GRU-based model framework for tool state recognition under unbalanced data.

As most of the tools in their full life cycle are in the normal wear state, the data under this state is far more than in the initial wear state or severe wear state of the data, generally regarded as the former as the majority class sample data, the latter as the minority class sample data, and there are differences in the amount of data in different wear states, so that the model training lacks sufficient minority class sample data, which in turn affects the model’s ability to generalize. At the same time, under the influence of electromagnetic interference, mechanical vibration, temperature change, and other factors, tool state data often contains a large number of noise components, which further increases the difficulty of data analysis and processing. In addition, with the application of GNN in the field of tool condition recognition, it is important to study how to convert the tool condition data into graph data. While the state data of tools is mostly sensor data deployed at different locations, there may be complex correlations, which cannot be adequately reflected when constructing graph data with a single metric. Furthermore, although GNNs perform well in efficiently capturing spatial dimension features of the data, their ability to mine temporal dimension features is lacking, a limitation that may hinder the full revelation of potential wear state information in the data, which in turn negatively affects the subsequent recognition of the tool state. Therefore, in this paper, a POGNN-GRU-based tool state recognition model framework under unbalanced data is designed, as shown in Figure 1, which mainly includes an unbalanced data processing stage and a state recognition stage. In the unbalanced data processing stage, for the problem of noise in multi-sensor data and data imbalance in different states, an adaptive noise removal strategy is introduced to identify and remove the noise points in the minority class samples, and based on the ideas of ADASYN and MWMOTE, the IMWMOTE oversampling method is designed to augment the data in the minority class samples and obtain the balanced dataset. In the stage of tool wear state recognition, a graph data construction method based on a multi-metric basis and a Gaussian kernel weight function is proposed to obtain lighter POG graph data through the process of constructing the base graph with a multi-metric basis, node edge connection pruning optimization, and a Gaussian kernel weight function for calculating the edge weights. On this basis, the POGNN-GRU model is constructed to extract the spatio-temporal features in the data for the classification of the graph and to realize intelligent recognition of tool wear state.

### 2.2. Imbalanced Data Processing

Oversampling methods can effectively mitigate the imbalance phenomenon of the data, reduce the sample bias, and improve the model’s ability to learn from it. For example, the ADASYN oversampling method can synthesize more sample instances of harder-to-learn samples through weight adjustment, which takes into account the differences between samples of different minority classes and can effectively alleviate the inter-class imbalance phenomenon of the data and the problem of sample aliasing, but does not adequately take into account the influence of boundary samples and outliers and is prone to phenomena such as over-generalization [12]. The MWMOTE oversampling method focuses on boundary samples that contain important information and are difficult to learn, and accordingly synthesizes more effective new samples, which can effectively alleviate the overgeneralization of synthetic samples and the problem of intra-class imbalance of data, but the number of samples generated for a few classes is mainly determined by human beings and lacks objectivity [13]. In addition, the presence of noise in the original data can introduce incorrect samples into the new dataset or make samples in certain regions over-synthesized, which in turn affects the generalization ability of the model. Currently, noise in samples is often handled with the help of the *k*-NN method, in which a sample is discriminated as noise for elimination if all *k* nearest neighbors of the sample belong to other sample categories [28,29]. Although the method reduces the noise effect to some extent, it may mistakenly retain the true noise as normal data or incorrectly exclude the critical boundary data (pseudo-noise). Therefore, this paper introduces the adaptive noise removal strategy and designs the IMWMOTE algorithm for mitigating the data imbalance problem and reducing the effect of noise based on ADASYN and MWMOTE ideas. The implementation details are as follows:

The k’-nearest neighborhood densities of the samples can also be used as the basis for noise discrimination [30]. Therefore, in order to identify and remove the noisy data in the sample more accurately, this paper introduces an adaptive noise removal strategy, that is, *k*-NN method, which is used to construct a set of suspected noise points $X_{n}$ and calculate the k’-nearest neighborhood densities of the suspected noise from $X_{n}$. On the basis of this, obtain the decision set D of the suspected noise, according to the following formula [29]:

(1) $$\begin{array}{l} D_{j}=1-w\times d_{j}^{S_{c}}\times (1-d_{j}^{B_{c}})\\ \;\;\;\;\;\;\;\;\;\;\;\;\;\;\;\;\;\;\;\;\;\;\;\;\;\;\;\;\;\;-(1-w)\times De_{j}^{S_{k}}\times (1-De_{j}^{B_{k}}),\\ \;\;\;\;\;\;\;\;\;\;\;\;\;\;\;\;\;\;\;\;\;\;\;\;\;\;\;\;\;\;j=1,2,\ldots ,N_{s} \end{array}$$

(2) $$D=\mathrm{sort}\{D_{j}\},j=1,2,\ldots ,N_{s}$$

where $j=0,2,\ldots ,N_{s}-1$, $N_{s}$ represents the number of suspected noise; $D_{j}$ is the decision value of the *j*th suspected noise, and the smaller the value is, the higher the probability that the corresponding suspected noise is true noise; $d_{j}^{S_{c}}$ and $d_{j}^{B_{c}}$ represent the Euclidean distance between the suspected noise $x_{j}\in X_{n}$ and the center of the minority class samples as well as majority class samples, respectively; $De_{j}^{S_{k}}$ and $De_{j}^{B_{k}}$ represent the k’-nearest neighborhood densities of minority class samples and the k’-nearest neighborhood densities of majority class samples of $x_{j}$, respectively; and w∈{0,0.1,…,1} is the weight factor of the Euclidean distance and the neighborhood densities.

To more accurately deal with the noise in the samples, take $j=0,2,\ldots ,N_{s}-1$, delete the first j suspected noise in D, and obtain a new minority sample set $X_{’}^{mi}$; subsequently, construct the imbalanced dataset $X^{ib}=[X_{ma};X_{mi}^{’}]$, $X_{ma}$ denotes the majority class sample set; take one of the minority class samples $x_{m}$, and achieve a synthesis of new samples by using the SMOTE method [10]; repeat the above steps until you obtain the balanced dataset $X^{b}$; use $X^{b}$ to train the classifier, then input the imbalanced dataset $X^{ib}$ into the trained model, and calculate the G-mean value denoted as $G_{j}$; finally, output the minority class sample set that corresponds to the maximum value of $G_{j}$ for the first time and denoted as $X_{mi}^{no}$ to be used for the subsequent synthesis of new samples.

In order to improve the ability of model to recognize the boundary between the minority class and the majority class and reduce the risk of overfitting, this algorithm is based on the MWMOTE idea to find the boundary samples between the majority class and the minority class and calculate the information weight $w_{in}(x_{i},x_{j})$, selection weight $W(x_{i})$ and sampling probability of different samples $P(x_{i})$ [13]. On this basis, considering the differences between different samples, this algorithm estimates the number of samples to be synthesized based on the ADASYN idea [12] and the calculated sample sampling probability. Subsequently, the synthesis of new samples is carried out to construct a balanced dataset. The specific implementation steps are shown in Algorithm 1.
**Algorithm 1**. IMWMOTE($X_{mi}^{no}$, $X_{ma}$, $k_{1}$, $k_{2}$, $k_{3}$, β)**Inputs**:$X_{mi}^{no}$: Noise-processed minority class sample dataset;$X_{ma}$: Majority sample dataset;$k_{1}$: The number of neighbors used to construct the set of majority class boundary samples;$k_{2}$: The number of neighbors used to construct the set of minority class boundary samples;$k_{3}$: The number of neighbors of minority class boundary samples;β: The proportion of majority and minority samples after sampling.**Procedure Begin:**For each minority class sample $x_{i}\in X_{mi}^{no}$, compute the $k_{1}$-nearest neighbor majority class samples set ($X_{k}^{ma}(x_{i})$), and obtain the set of majority class boundary samples $X_{b}^{ma}=\{X_{k}^{ma}(x_{i})|x_{i}\in X_{mi}^{no}\}$. Similarly, compute the $k_{2}$-nearest neighbor minority samples set ($X_{k}^{mi}(x_{j})$) for each $x_{j}\in X_{b}^{ma}$, and obtain the set of minority class boundary samples $X_{b}^{mi}=\{X_{k}^{mi}(x_{j})|x_{j}\in X_{b}^{ma}\}$.2.For each $x_{j}\in X_{b}^{ma}$ and $x_{i}\in X_{b}^{mi}$, use the formula $w_{in}(x_{i},x_{j})=f_{cl}(x_{i},x_{j})\cdot f_{de}(x_{i},x_{j})$ to compute the information weight. The calculation of $f_{cl}(x_{i},x_{j})$ and $f_{de}(x_{i},x_{j})$ can be found in reference [13].3.For each $x_{i}\in X_{b}^{mi}$, compute the selection weight $W(x_{i})=\sum_{x_{j}\in X_{b}^{ma}}w_{in}(x_{i},x_{j})$ and the probability of sampling $P(x_{i})=W(x_{i})/\sum_{x_{i}\in X_{b}^{mi}}W(x_{i})$.4.For the sample $x_{i}$, calculate the proportion of majority class samples in its $k_{3}$-nearest neighbors by using $\alpha_{i}=B/k_{3}$, where B denotes the number of majority class samples in the $k_{3}$-nearest neighbors of $x_{i}$. Then, use the formula $\bar{\alpha_{i}}=\alpha_{i}/\sum_{i=1}^{N_{bm}}\alpha_{i}$ to standardize, where $N_{bm}$ denotes the number of samples in $X_{b}^{mi}$.5.Calculate synthesis ratio $c_{i}=\lambda \cdot P(x_{i})+(1-\lambda )\cdot \bar{\alpha_{i}},\;\lambda =0.5$, and $\bar{c_{i}}=c_{i}/\sum_{i=1}^{N_{bm}}c_{i}$.6.Calculate the number of samples $n_{i}^{*}$ that need to be synthesized for $x_{i}$ through the formula $n_{i}^{*}=\bar{c_{i}}\times N$, where $N=\beta (N_{ma}-N_{mi’})$, β∈(0,1], $N_{ma}$ denotes the number of samples of the majority class, and $N_{mi’}$ denotes the number of samples of $X_{mi}^{no}$.7.Initialize the dataset such that $X_{mi}^{0}=X_{mi}^{no}$, and perform AHC clustering on $X_{mi}^{no}$ to generate M subclusters $Sub_{1},Sub_{2},\ldots ,Sub_{M}$.*Do for* $i=1,\ldots ,N_{bm}$(1)Take a sample $s_{i}$ from $X_{b}^{mi}$, while $s_{i}\in Sub_{m},m=1,2,\ldots ,M$.(2)*Do for* $j=1,\ldots ,n_{i}^{*}$Take another sample q from $Sub_{m}$ randomly, and synthesize the new sample according to the formula $x_{ne}=s_{i}+\mu (q-s)$, where μ∈[0,1].Update the dataset $X_{mi}^{0}$ such that $X_{mi}^{0}=X_{mi}^{0}\cup \{x_{ne}\}$.*        End Loop* *  End Loop*
  Store the completed sampled dataset as $X_{mi}^{ne}$.8.Obtain the balanced dataset $X_{b}=[X_{ma};X_{mi}^{ne}]$.**END****Output:** Balanced dataset $X_{b}$ after IMWMOTE sampling.

In summary, the IMWMOTE algorithm is important for improving the accuracy of tool state recognition by adaptively removing the noise in the minority class of faulty samples and performing data augmentation on the minority class of samples in order to alleviate the problem of intra-class imbalance between data classes.

### 2.3. POGNN-GRU Model Construction

**Figure 2:**
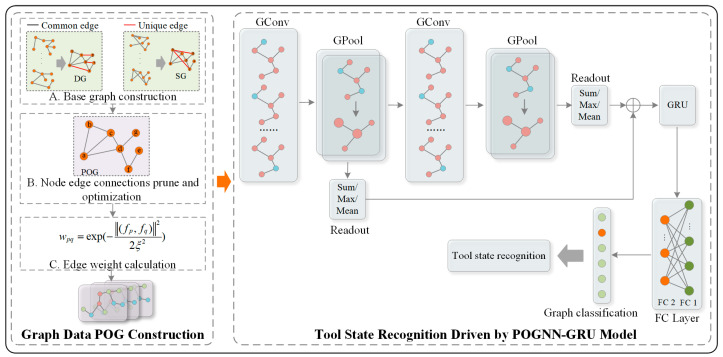
POGNN-GRU model framework.

Compared with other deep learning methods, GNN treats nodes as interconnected entities and considers topological information in them, while GNN can capture the unique features of each node in the graph with the help of edge connectivity and node neighborhood, which is widely applicable to solve node-level, edge-level, and graph-level tasks [31]. Although GNN shows better performance in aggregating and extracting spatial dimension features from data, it is unable to comprehensively and efficiently extract temporal dimension features, which is not conducive to subsequent tool state recognition. GRU is a special recurrent neural network structure that effectively solves the gradient vanishing and gradient exploding problems that the traditional recurrent neural network faces when dealing with long sequence data by introducing the mechanisms of reset and update gate problems, and thus can better capture the long-term dependencies in the data [32]. Therefore, the POGNN-GRU model shown in Figure 2 is constructed to extract the state feature information from the sensor data.

The model mainly consists of two parts: POG graph data construction and POGNN-GRU-driven tool state recognition. The former mainly contains the base graph construction, pruning optimization of node edge connections, and edge weight calculation. The latter mainly contains two GConv layers, two GPool layers, two readout layers, one GRU layer, and two FC layers. Among them, the GConv layer is used to learn the feature representations of nodes; the GPool layer achieves feature dimensionality reduction by aggregating multiple node features to form a graph-level feature representation; the Readout layer integrates the node representations in subgraphs using Sum/Max/Mean operations and introduces the residual linkage mechanism to alleviate the problem of gradient vanishing; the GRU layer is designed to extract the time–dimensional features embedded in data; and the FC layer is designed to extract the time–dimensional features contained in data. dimension features; and the FC layer is used for feature fusion and the final task-specific output for graph classification to achieve tool state recognition. The implementation details are as follows:

The state data of a tool is usually multi-sensor data with complex spatial correlations among them, which can be presented in the form of a graph. High-quality graph data representation enables GNNs to learn the topology and complex relationships between nodes in the graph more effectively, thus improving the performance and generalization ability of the model. Therefore, for sensor data with different feature scales, in this paper, we take the Mahalanobis distance and cosine similarity as the measurement basis, construct multiple distance base graphs $G_{d}$ and similarity base graphs $G_{s}$ with different thresholds to capture multilevel information, and perform weighted fusion of base graphs of the same type to obtain the corresponding types of weighted graphs, which are denoted as DG and SG, respectively. Subsequently, based on the similar structure of the two types of graph data, the node-to-node edge connectivity relationships are pruned and optimized, and edge weights are calculated with Gaussian kernel weight function to construct POG graph data.

Specifically, intercept the IMWMOTE-processed dataset with a sliding window to obtain subsamples $x_{i}\in R^{n\times m}$ of the same size, where n denotes the number of sensors and m denotes the length of the subsample data. Each subsample usually consists of multiple sensor data points, and each sensor can be regarded as a node in the graph when the graph data are constructed. Subsequently, use the Mahalanobis distance [33] to measure the distance between the node features $f_{p}$ and $f_{q}$:

(3) $$M\_dis_{pq}=\sqrt{{\left(f_{p}-f_{q}\right)}^{T}S^{-1}(f_{p}-f_{q})}$$

where S is the covariance matrix of the subsample.

Use the calculated Mahalanobis distance and different thresholds to find the nearest neighbor for each node and to establish the edge-connectivity relationship between them so as to construct the distance base graph $G_{d}$. At the same time, refer to Equation (9), calculate the cosine similarity between nodes, and when the cosine similarity $Sim_{pq}$ of node features $f_{p}$ and $f_{q}$ is greater than the specified threshold, establish the edge connection of two nodes so as to construct the similarity base graph $G_{s}$.

(4) $$Sim_{pq}=\frac{f_{p}\cdot f_{q}}{\left\|f_{p}\right\|\cdot \left\|f_{q}\right\|}$$

On this basis, different types of base graphs are weighted and fused to obtain the corresponding weighted graphs, DG and SG, respectively. The two base graphs constructed above have global similarity in node-edge connections but have local differences. In order to increase the validity of the constructed data edge connection, prune the “unique edges” as shown in Figure 2. Finally, use the Gaussian kernel weight function to calculate the edge weight $w_{pq}$ between the connected nodes to achieve the construction of graph data. In POG, the specific formula is defined as follows [24], where ξ represents the Gaussian kernel bandwidth:

(5) $$w_{pq}=\exp(-\frac{{\left\|\left(f_{p},f_{q}\right)\right\|}^{2}}{2\xi^{2}})$$

Perform the same operation on each subsample of data in the time dimension to construct the POG spatio-temporal graph, which provides data support for the subsequent training and validation of the model.

In the process of POGNN-GRU-driven tool state recognition model construction, this paper chooses ChebyNet as the GConv layer and EdgePool as the GPool layer [34,35]. Among them, ChebyNet is a GNN model based on graph convolution theory, which implements the feature decomposition of the Laplace matrix of the graph through Chebyshev polynomial approximation to capture the global information of the graph, thus reducing the computational complexity and the number of parameters of the model. The formula for graph convolution can be expressed as follows:

(6) $$\begin{array}{l} x*f_{\theta }=Uf_{\theta }(\Lambda )U^{T}x=U\sum_{j=0}^{K}\theta_{j}\Lambda^{j}U^{T}x\\ =\sum_{j=0}^{K}\theta_{j}(U\Lambda U^{T}{)}^{j}x=\sum_{j=0}^{K}\theta_{j}L^{j}x \end{array}$$

where ∗ denotes convolution operation; *x* denotes the input features; $f_{\theta }$ is the convolution kernel parameterized by θ; *U* denotes the matrix consisting of the eigenvectors of *L*; Λ denotes the matrix consisting of the eigenvalues of *L*.

ChebyNet approximates the graph convolution formula based on Chebyshev polynomials as follows:

(7) $$x*f_{\theta }=\sum_{j=0}^{K}\theta_{j}L^{j}x\approx \sum_{j=0}^{K}\theta_{j}T_{j}(\bar{L})x$$

where $\lambda_{\max}$ denotes the largest eigenvalue of the graph Laplace matrix and $I_{n}$ denotes the identity matrix; $T_{j}(\bar{L})=2\bar{L}T_{j-1}(\bar{L})-T_{j-2}(\bar{L})$ denotes the Chebyshev polynomials of order *j*, $T_{1}(\bar{L})=\bar{L}=2L/\lambda_{\max}-I_{n}$, $T_{0}(\bar{L})=I_{n}$.

The EdgePool method is able to aggregate the nodes connected by an edge into a new node by iteratively selecting the edge and performing a contraction operation, maintaining the connection relationship between the original nodes in the process. This can be achieved using the following formula:

(8) $$\left\{\begin{array}{l} S_{row}(e_{ij})=\varpi \cdot (c_{i}||c_{j})+\eta \\ score_{ij}=0.5+softmax(S_{row}(e_{ij})) \end{array}\right.$$

where $e_{ij}$ represents the edge between the *i*th and *j*th node; $S_{row}(e_{ij})$ represents the row score; $c_{i}$ and $c_{j}$ represent the node features; ϖ and η represent the learnable parameters; and $score_{ij}$ represents the final score after normalization operation on all edges of the node.

## 3. Case Study

### 3.1. Dataset Description

**Figure 3:**
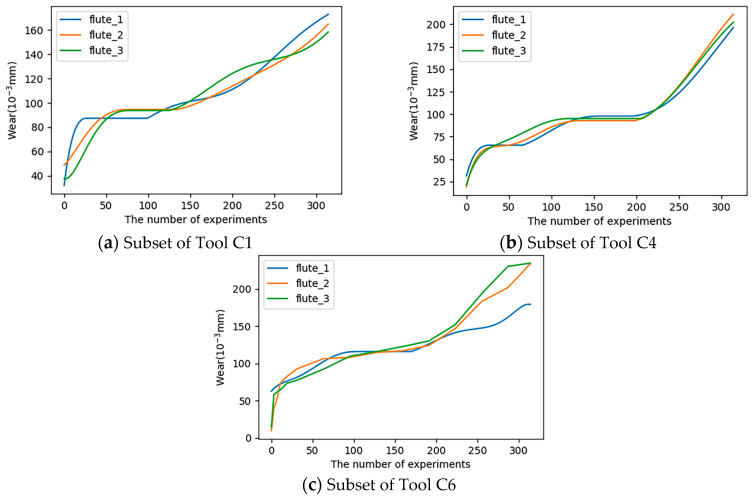
(**a**) Wear variation curves of tools C1; (**b**) wear variation curves of tools C4; (**c**) wear variation curves of tools C6.

In order to verify the performance of the model proposed in this paper, the PHM2010 dataset [36] and the High-speed milling of thin-walled parts (HMoTP) dataset [37] were used in this experiment. The PHM2010 dataset contained the results of the CNC milling machine using a 6 mm triple-fluted tungsten carbide ball head for milling of stainless steel (HRC52) workpiece, and the data collected by deploying seven kinds of sensors such as dynamometer, unidirectional accelerometer, acoustic emission and so on during the work of CNC milling machine, specifically containing the force data, vibration data and an AE-RMS data under three different directions, and the sampling of the collected data was realized by the DAQ-NI PCI1200, and the above operation was repeated for the six tools respectively to obtain the six sub-datasets of C1, C2, C3, C4, C5, C6 six sub-datasets, where the C1, C4, C6 sub-datasets also contained measurements of tool wear obtained using a LEICA MZ12 microscope (LEICA, Wetzlar and Mannheim, Germany), so two of these sub-sets were selected as training sets and the other as a test set. The wear variation curves of tools C1, C4, and C6 are shown in Figure 3.

Each subset of C1, C4, and C6 contained sensor data from 315 cycles of experiments. Figure 3 shows the wear change curves of tools C1, C4, and C6. It could be seen from the figure that the wear degree of tools was relatively low during the first 20 cycles of experiments, and this stage was regarded as the stage of slight tool wear and was represented as Slight_Wear. During 20–200 cycles, the tool wear degree increased, but the degree of change was small. This stage was regarded as the intermediate tool wear stage, which was expressed as Medium_Wear. However, the degree of tool wear increased rapidly after 200 cycles of experiments, and this stage was regarded as the stage of severe tool wear, which was expressed as Severe_Wear.

**Figure 4:**
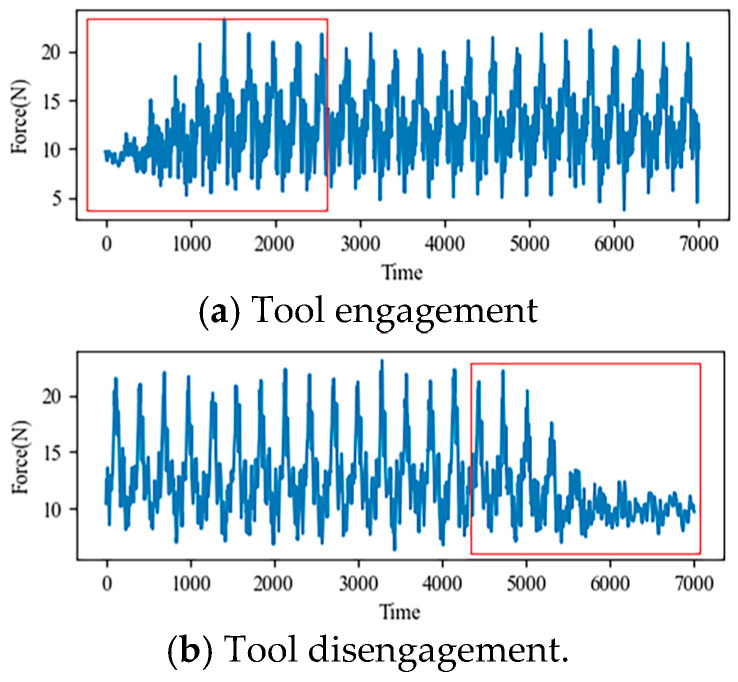
(**a**) Partial force data for single-cycle experiments during tool engagement; (**b**) Partial force data for single-cycle experiments during tool disengagement.

For the single cycle experiment, since no cutting operation was carried out on the workpiece in the process of tool engagement and tool disengagement (as shown in the red box in Figure 4, which displays a portion of the force data collected by a sensor during the above processes), the tool was not worn at this time, so the data collected in the process of tool engagement and tool disengagement in each cycle experiment was regarded as invalid data to be eliminated.

Subsequently, a non-overlapping sliding window of length 1024 and the Min-Max normalization method were used to sub-sample and normalize the remaining data to eliminate the effect of magnitude between different data features. Then, construct the unbalanced datasets Medium_Wear/Slight_Wear and Medium_Wear/Severe_Wear as the experimental data for IMWMOTE. On this basis, the multivariate time series of every 10 subsamples in the dataset were transformed into one graph to realize the construction of the graph dataset.

The HMoTP dataset contained the milling experimental data of three sets of tools consisting of one tool holder and two inserts under the full life cycle, during which the spindle speed was 8000 rpm, the cutting speed was 351.85 m/min, the feed rate was 1280 mm/min, the radial depth of cut was 0.2 mm, and the axial depth of cut was 4 mm. The experiment was conducted by installing a triaxial accelerometer, Dytran 3263A1, and a Kistler rotary dynamometer to obtain the vibration signals, milling force signals, and axial bending moment signals on the x-axis, y-axis, and z-axis of a tool machining a thin-walled part. Three sets of tools each had 100 cutting data files, which made up the three subdatasets, T01, T02, and T03, and two of them were selected as the training set and the other as the test set.

**Figure 5:**
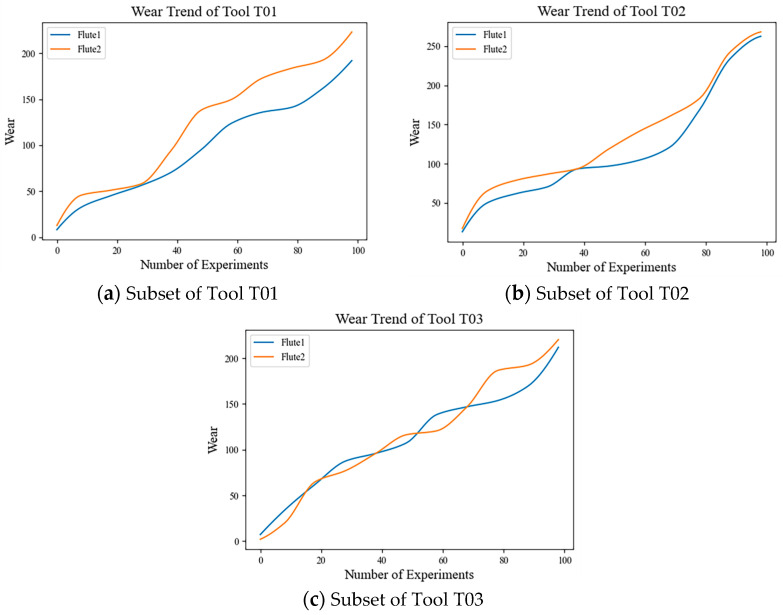
(**a**) Wear variation curves of T01; (**b**) wear variation curves of T02; (**c**) wear variation curves of T03.

Each cutting data corresponds to a tool wear value, composing ToolWeaT01, ToolWeaT02, and ToolWeaT03 tool wear data, and the corresponding wear change curves are shown in Figure 5. As shown in the figure, the first 20 cycles of the experiment tool wear degree were low, could be regarded as a slight wear stage, and denoted as Slight_Wear; in 20–80 cycles of the experiment tool wear degree increased, and the trend of change compared with the first stage became slower; the stage could be regarded as the tool intermediate wear stage, and denoted as Medium_Wear; the trend of change in 80–100 cycles of the experiment wear further changed; the stage was regarded as the severe tool wear stage, and denoted as Severe_Wear. Subsequently, use a non-overlapping sliding window with a length of 256 to divide the subsamples, and the rest of the data processing is consistent with PHM 2010.

### 3.2. Objective Function and Network Model Parameterization

In this paper, we use two FC layers as the output layer of the model to generate a probability distribution for each state category and utilize the Cross-Entropy (CE) loss as the objective function for the classification task.

(9) $$Loss_{CE}=-\sum_{j=1}^{C}r_{j}\log(p_{i})$$

where *C* denoted the number of state types; *r_j_* denoted the sample label, *j* = 1,2,…, *C*; *p_i_* denoted the probability of being in the *i*th state.

We ran this experiment on Windows 11 operating system with AMD Ryzen 7 5800H processor (AMD, Santa Clara, CA, USA), 16 GB of running memory, NVIDIA GeForce RTX 3050 Laptop GPU (NVIDIA, Santa Clara, CA, USA) for graphics, and Python 3.10.11 as well as Pytorch 2.1.0+cu121 and PyTorch Geometrics 2.4.0, to construct the model proposed in this paper.

To achieve tool state recognition, we constructed the POGNN-GRU model and used the Adam optimizer. Furthermore, the initial learning rate was set to 0.001, the weight decay value was initialized to $5\times 10^{-4}$, the Dropout was set to 0.5, the batch size for model training was set to 64, the epoch was set to 100, and the remaining model parameters were set under the PHM 2010 and HMoTP datasets, respectively, as shown in Table 1.

### 3.3. Model Metrics

As shown in Table 2, there are the following four results for model classification, and in order to reasonably evaluate the performance of the model, appropriate measurement indicators need to be used. The model measurement and evaluation indexes used in this paper mainly contain accuracy, precision, recall, and F1 (F1-score), which are calculated as follows.

(10) $$\left\{\begin{array}{l} \mathrm{Acc}=(\mathrm{TP}+\mathrm{TN})/(\mathrm{TP}+\mathrm{TN}+\mathrm{FP}+\mathrm{FN})\\ \mathrm{Pre}=\mathrm{TP}/(\mathrm{TP}+\mathrm{FP})\\ \mathrm{Rec}=\mathrm{TP}/(\mathrm{TP}+\mathrm{FN})\\ F1=2\times \mathrm{Precision}\times \mathrm{Recall}/\left(\mathrm{Precision}+\mathrm{Recall}\right) \end{array}\right.$$

### 3.4. Analysis of Experimental Results

**Figure 6:**
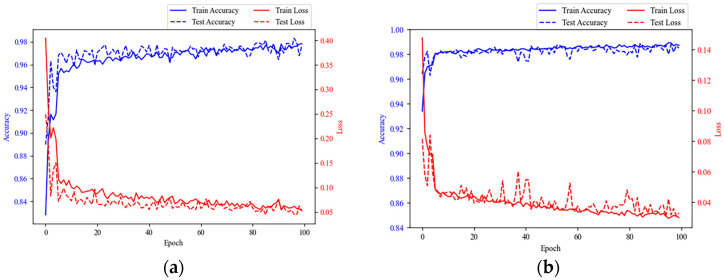
Model training and testing results; (**a**) the result of PHM2010 dataset; (**b**) the result of HMoTP dataset.

Input the training set and test data under the PHM2010 dataset and HMoTP dataset into the proposed model in this paper, respectively, and the corresponding accuracy and loss function values were shown in Figure 6, which showed that the model gradually tended to be stable after about 20 epochs of training, and the accuracy and loss function values were close to 98% and 0, respectively, indicating that the model did not suffer from serious overfitting or underfitting phenomena, and it had good classification and generalization abilities.

**Figure 7:**
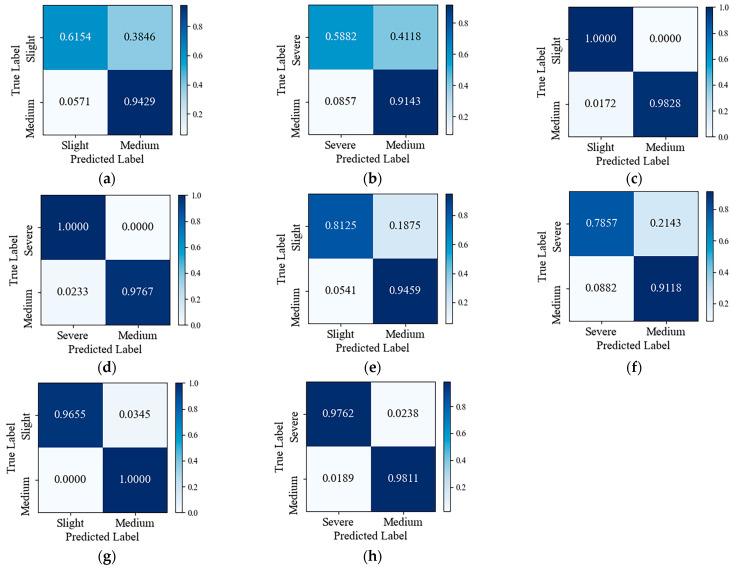
Confusion Matrix, (**a**) Medium_Wear/Slight_Wear classification (no sampling) under PHM2010 dataset; (**b**) Medium_Wear/Severe_Wear classification (no sampling) under PHM2010 dataset; (**c**) Medium_Wear/Slight_Wear classification (with IMWMOTE) under PHM2010 dataset; (**d**) Medium_Wear/Severe_Wear classification (with IMWMOTE) under PHM2010 dataset; (**e**) Medium_Wear/Slight_Wear classification (no sampling) under HMoTP dataset; (**f**) Medium_Wear/Severe_Wear classification (no sampling) under HMoTP dataset; (**g**) Medium_Wear/Slight_Wear classification (with IMWMOTE) under HMoTP dataset; (**h**) Medium_Wear/Severe_Wear classification (with IMWMOTE) under HMoTP dataset.

The confusion matrix could visualize the performance ability of the model in each category. In order to analyze the impact of whether the number of samples in the dataset was balanced or not on the model recognition results, this paper used the confusion matrix to show the results of the POGNN-GRU model before and after data balance under the PHM2010 dataset and the HMoTP dataset, as shown in Figure 7. Figure 7a,b,e,f showed the results before data set balancing, and Figure 7c,d,g,h showed the results after data set balancing, and it could be seen that the model under the balanced dataset could more accurately identify the wear state of the tool.

To verify the performance of the model in recognizing tool states under data imbalance conditions, the imbalanced datasets Medium_Wear/Slight_Wear and Medium_Wear/Severe_Wear were processed by ADASYN, MWMOTE, and IMWMOTE, respectively, and compared with the results of the unprocessed (No-Sampling). Since the model was more inclined to predict the majority class under the unbalanced dataset, the misclassification of the minority class samples had less impact on the overall accuracy, and a higher accuracy may also be obtained. Furthermore, the F1 value took into account the model’s accuracy and recall ability, so the F1 value was adopted here as the model performance measure. In this process, RMS features were extracted from the processed dataset as input to the SVM classifier.

The average statistical results after six trials with different methods were presented in Table 3, from which it could be seen that the performance of the model after using ADASYN was slightly worse than the model performance of No-Sampling under both datasets regarding the Medium_Wear/Slight_Wear and Medium_Wear/Severe_Wear state recognition of the tools. In contrast to the MWMOTE and IMWMOTE methods, which dealt with noise in the minority classes of samples, the F1 values of the models both improved for Medium_Wear/Slight_Wear and Medium_Wear/Severe_Wear state recognition compared with No-Sampling. Furthermore, compared with MWMOTE, the model with the IMWMOTE method had higher F1 values with 9.10%, 9.34%, 1.14%, and 4.09% improvement, respectively.

The above results illustrated that the noise in the tool wear dataset adversely affected the performance of the classifier. Furthermore, the IMWMOTE method proposed in this paper outperformed the other methods in different unbalanced datasets, which indicated that the method was able to effectively remove the noise in the minority class samples to alleviate the unbalanced problem of the data and was more suitable for practical tool wear application monitoring.

To further explore the performance of the proposed model in this paper, the IMWMOTE processed datasets were used to construct the POG graph data, and GCN [25], GAT [24], ChebyNet [34], LSTM [17], GRU [18], HoGNN [38], MRF-GCN [39], and A3T-GCN [40] were used as the baseline methods. The average statistical results after six experiments for different models under the PHM 2010 dataset and the HMoTP dataset are shown in Table 4 and Table 5, respectively. The experimental results in the table showed that GRU had better performance compared with LSTM for Medium_Wear/Slight_Wear and Medium_Wear/Severe_Wear state recognition, indicating that the GRU model on the tool dataset captures the dependencies in the time series better. In addition, ChebyNet had higher accuracy in tool state recognition compared with other GNN models, indicating that ChebyNet had better feature extraction ability than other GNN models. And compared with the ChebyNet and GRU models, the POGNN-GRU model had improved in each index. Furthermore, when performing Medium_Wear/Slight_Wear state recognition under the PHM 2010 dataset, the accuracy, precision, recall, and F1 values of the POGNN-GRU model were 98.11%, 98.85%, 97.66%, and 98.25%, respectively, which were improved in comparison to the optimal baseline model MRF-GCN by 1.62%, 2.09%, 1.29%, and 1.69%, respectively. For Medium_Wear/Severe_Wear state recognition, the accuracy, precision, recall, and F1 values of the POGNN-GRU model were 97.44%, 98.78%, 97.15%, and 97.96%, respectively, which were 1.17%, 3.66%, 0.43%, and 2.03% higher than the optimal baseline model, A3T-GCN, respectively. When performing Medium_Wear/Slight_Wear and Medium_Wear/Severe_Wear state recognition under the HMoTP dataset, the model proposed in this paper also achieved the best results compared with other models. The above results indicated that the use of the model proposed in this paper to mine the spatio-temporal information in the state data was of great significance in reducing the misidentification of tool states.

**Figure 8:**
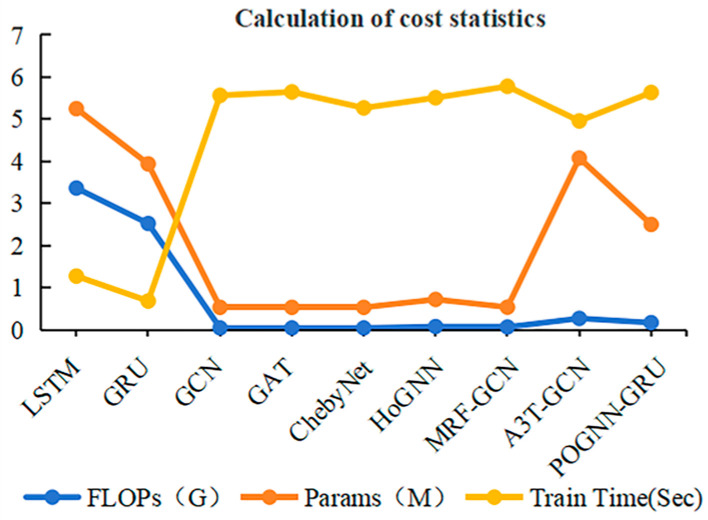
The computational cost of different models.

In addition, this paper evaluated the computational cost of each model by the Floating Point Operations Per Second (FLOPS), the number of parameters (Params), and the training time (the time required for each epoch of the model training process). Shown in Figure 8 is the computational cost of each model under the PHM 2010 dataset (the specific data are shown in Table 6). It could be seen that the GNNs model as well as the HoGNN and MRF-GCN models had smaller FLOPS and Params compared with the LSTM and GRU, but the corresponding training time was longer. The A3T-GCN model had smaller FLOPS compared with LSTM and GRU, while its Params were larger compared with GRU. The models used in this paper showed a smaller increase in FLOPS and training time compared with the GNNs, HoGNN, and MRF-GCN models, and the corresponding FLOPS were smaller than those of the LSTM and GRU models. In addition, although the Params of the models used in this paper increased compared with those of the GNNs, HoGNN, and MRF-GCN models, they decreased by 52.36%, 36%, and 38.65%, respectively, compared with the LSTM, GRU, and A3T-GCN models.

### 3.5. Ablation Experiments

**Figure 9:**
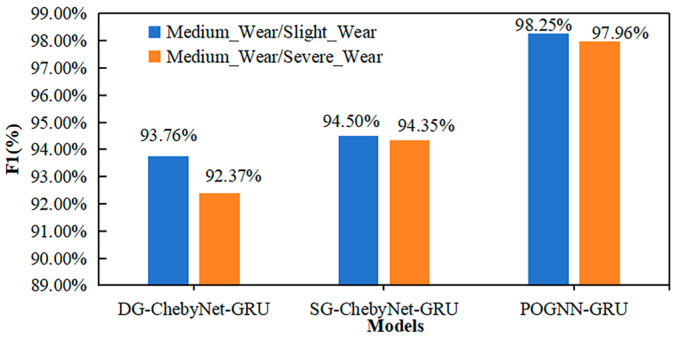
The result of ablation experiments 1.

In order to further investigate the effects of different graph data construction methods, the ChebyNet module and the GRU module, on the model performance, two ablation experiments were designed under the PHM 2010 dataset in this paper for validation analysis. Ablation experiment 1 used DG, SG, and the POG method proposed in this paper for graph data construction, respectively, and the results are shown in Figure 9. DG and SG were based on a single metric to determine the edge connection relationship of graph nodes, respectively, and the result of DG was lower than that of SG for both datasets. POG was based on a multi-scale multi-metric basis to determine the edge connection relationship of graph nodes, and the Gaussian kernel weight function was utilized to calculate the edge weights. The results of the corresponding model were optimal, indicating that the graph data construction method proposed in this paper further improved the information expression of topology.

The ablation experiment 2 adopted POG as the graph data construction method and introduced different GNN models, as well as LSTM and GRU models, respectively. The experimental results are shown in Table 7 below. It could be seen that, compared with the GNNs model that only extracts features in the spatial dimension and the LSTM as well as the GRU models that only extract features in the temporal dimension in Table 4 and Table 5, extracting features from multiple dimensions could further improve the accuracy of model classification. In addition, ChebyNet and LSTM improved their model performance compared with other models.

## 4. Conclusions

In this paper, we propose a tool state recognition method based on POGGNN-GRU under unbalanced data, process the unbalanced data through IMWMOTE, on the basis of which we construct a POGNN-GRU model to deeply mine the spatio-temporal dependence between data to realize tool state recognition, and experimentally validate it on PHM2010 and the HMoTP dataset. The main conclusions of the study are as follows:

- The IMWMOTE unbalance data processing algorithm proposed in this paper can effectively improve the unbalance phenomenon between and within classes under different wear states of the tool, largely reduce the impact of noise in minority classes of samples on tool state recognition, and improve the overall noise immunity and accuracy of the model.
- In this paper, we propose a graph data construction method that introduces a graph data pruning optimization strategy based on a multi-scale and multi-metric basis in the graph construction process. This optimization strategy further refines the topological information and improves the topological representation, which is important for improving the model performance.
- The POGNN-GRU model proposed in this paper utilizes a graph data pruning optimization strategy based on a multi-scale and multi-metric basis for graph data construction and extracts node features from multi-dimensions to mine the spatio-temporal information embedded in the tool state data to achieve tool wear state recognition. The model validation is carried out with the PHM 2010 and HMoTP datasets, and the results show that the highest accuracy, precision, recall, and F1 values of the proposed model are 98.11%, 98.85%, 97.66%, 98.25%, 97.44%, 98.78%, 97.15%, and 97.96% for state recognition under the PHM 2010 dataset. Furthermore, the model achieves the highest accuracy of 98.40% and 97.68% under the HMoTP dataset, proving the feasibility and effectiveness of the proposed model in this paper.

## Figures and Tables

**Table 1:** Model parameter setting.

Layer	PHM 2010 Parameter Settings	HMoTP Parameter Settings
GConv 1	1024 × 1024	256 × 256
BatchNorm 1	1024	256
EdgePool 1	1024	256
Readout 1	Sum	Sum
GConv 2	1024 × 1024	256 × 256
BatchNorm 2	1024	256
EdgePool 2	1024	256
Readout 2	Sum	Sum
GRU	1024 × 512	256 × 128
FC 1	512 × 256	128 × 128
FC 2	256 × C	128 × C

**Table 2:** Model classification results.

Layer	Actual Positive	Actual Negative
Predicted Positive	True Positive, TP	False Positive, FP
Predicted Negative	False Positive, FN	True Positive, TN

**Table 3:** F1 statistics (%) of state recognition results under different oversampling methods.

Model	PHM2010 Dataset		HMoTP Dataset	
	Medium_Wear/ Slight_Wear	Medium_Wear/ Severe_Wear	Medium_Wear/ Slight_Wear	Medium_Wear/ Severe_Wear
No-Sampling	82.67	43.51	86.89	84.41
ADASYN [12]	71.07	75.39	86.14	82.73
MWMOTE [13]	83.26	81.06	91.27	87.53
IMWMOTE	90.84	88.63	92.31	91.11

**Table 4:** Statistics of different model tool state recognition results under PHM 2010 dataset.

Model	Medium_Wear/Slight_Wear				Medium_Wear/Severe_Wear			
	Acc (%)	Pre (%)	Rec (%)	F1 (%)	Acc (%)	Pre (%)	Rec (%)	F1 (%)
GCN [25]	90.77	89.32	93.12	91.18	89.09	90.12	91.13	90.62
GAT [24]	88.57	86.53	92.02	89.19	87.93	88.92	89.54	89.23
ChebyNet [34]	92.31	92.78	93.52	93.15	89.55	91.38	90.90	91.14
LSTM [17]	89.80	90.11	93.00	91.53	88.89	87.70	91.88	89.74
GRU [18]	90.57	92.58	92.50	92.54	89.23	90.66	90.16	90.41
HoGNN [38]	95.05	92.75	94.77	93.75	94.88	93.35	93.89	93.62
MRF-GCN [39]	96.55	96.83	96.41	96.62	95.59	95.41	94.57	94.99
A3T-GCN [40]	95.95	92.31	95.24	93.75	96.31	95.29	96.74	96.01
POGNN-GRU	98.11	98.85	97.66	98.25	97.44	98.78	97.15	97.96

**Table 5:** Statistics of different model tool state recognition results under HMoTP dataset.

Model	Medium_Wear/Slight_Wear				Medium_Wear/Severe_Wear			
	Acc (%)	Pre (%)	Rec (%)	F1 (%)	Acc (%)	Pre (%)	Rec (%)	F1 (%)
GCN [25]	92.74	88.34	95.07	91.58	91.33	92.44	89.72	91.06
GAT [24]	92.60	90.12	94.27	92.15	92.61	92.96	90.38	91.65
ChebyNet [34]	93.27	94.25	92.88	93.56	93.05	95.75	93.77	94.75
LSTM [17]	91.59	91.88	92.87	92.37	92.21	96.62	92.95	94.75
GRU [18]	92.01	94.16	93.80	93.98	92.55	91.97	93.38	92.67
HoGNN [38]	96.60	92.80	92.36	92.58	95.26	94.47	94.53	94.5
MRF-GCN [39]	95.35	95.98	95.52	95.75	95.83	93.382	96.36	94.85
A3T-GCN [40]	96.60	97.87	93.72	95.75	96.82	94.35	96.11	95.22
POGNN-GRU	98.40	100.00	97.75	98.86	97.68	97.19	99.33	98.25

**Table 6:** The computational cost of different models.

Model	FLOPs (G)	Params (M)	Train Time (s)
GCN [25]	0.0446	0.534	5.5666
GAT [24]	0.0446	0.534	5.6487
ChebyNet [34]	0.0446	0.534	5.2690
LSTM [17]	3.366	5.252	1.2763
GRU [18]	2.525	3.939	0.6842
HoGNN [38]	0.0787	0.7220	5.5110
MRF-GCN [39]	0.0666	0.5381	5.7812
A3T-GCN [40]	0.2719	4.0783	4.9594
POGNN-GRU	0.1709	2.5020	5.6348

**Table 7:** F1 statistics (%) for ablation experiment 2.

Model	Medium_Wear/Slight_Wear	Medium_Wear/Severe_Wear
GCN-LSTM [41]	94.83	94.27
GAT-LSTM [42]	94.70	92.84
ChebyNet-LSTM	95.02	93.31
GCN-GRU [43]	95.02	94.32
GAT-GRU [44]	94.97	94.10
POGNN-GRU	98.25	97.96

## Data Availability

The data supporting the findings of this study are available within the article. The experiments conducted in this research are based on the PHM 2010 and HMoTP datasets, which are the publicly available benchmark datasets for tool wear monitoring. The PHM 2010 dataset can be accessed at https://www.phmsociety.org/competition/phm/10 (accessed on 21 July 2024), and the HMoTP dataset can be accessed at https://runqiong.wang/dataset/ (accessed on 21 July 2024).
